# Comparison of the Risk of Pneumonia Between Fluticasone Furoate/Umeclidinium/Vilanterol and Multiple-Inhaler Triple Therapy in Patients with COPD Using Health Insurance Claims Data: Final Analysis of Post-Marketing Database Surveillance in Japan

**DOI:** 10.3390/jcm14134697

**Published:** 2025-07-02

**Authors:** Shoko Akiyama, Kenji Oda, Hiroko Mizohata, Natsuki Sasakura, Kenichi Hashimoto, Hiroki Maruoka

**Affiliations:** 1 Real World Data Analytics, Japan Development, GSK, Tokyo 107-0052, Japan; shoko.2.akiyama@gsk.com (S.A.); 2 Global Real-World Evidence & Health Outcomes Research Japan, GSK, Tokyo 107-0052, Japan; 3 Respiratory Medical Affairs, Japan Medical Affairs, GSK, Tokyo 107-0052, Japan

**Keywords:** chronic obstructive pulmonary disease, pneumonia, fluticasone furoate/umeclidinium/vilanterol, multiple-inhaler triple therapy, single-inhaler triple therapy, Japan

## Abstract

**Background/Objectives:** Due to limited current evidence, this post-marketing database surveillance study aimed to investigate the occurrence of hospitalization due to community-acquired pneumonia (CAP) among patients with chronic obstructive pulmonary disease in Japan who received single-inhaler triple therapy (fluticasone furoate/umeclidinium/vilanterol; FF/UMEC/VI) or multiple-inhaler triple therapy (MITT). **Methods:** This retrospective cohort study used health insurance claims data from the Medical Data Vision Co., Ltd. database (November 2017–April 2023) to identify overall and incident users of FF/UMEC/VI or MITT. Index date was the start of FF/UMEC/VI or MITT. Hazard ratios (HRs) for CAP hospitalization were assessed using inverse probability of treatment weighting based on propensity scores (PS). Incidence rates and time to occurrence of CAP hospitalization were also assessed. Adjustments were made to the PS model to address missing body mass index data. **Results**: In total, 8790 and 10,881 patients were included in the overall FF/UMEC/VI and MITT cohorts, and 3939 and 4017 patients were included in the incident FF/UMEC/VI and MITT cohorts, respectively. HR for CAP hospitalization among incident users ranged from 1.05 to 1.15 across all PS adjustments. Similar incidence rates of CAP hospitalization were reported among both cohorts and across all adjustments. The cumulative adjusted incidence rates of first CAP hospitalization at 360 days post-index among incident users was 0.060 and 0.054 in the FF/UMEC/VI and MITT cohorts, respectively. **Conclusions**: There was no difference in the risk of CAP between patients treated with FF/UMEC/VI and MITT. This safety information may help healthcare providers select appropriate treatments.

## 1. Introduction

Chronic obstructive pulmonary disease (COPD) is characterized by progressive reduced airflow [1] and has been shown to reduce patients’ quality of life [2]. COPD is also a risk factor for community-acquired pneumonia (CAP) [3]; older age (>65 years) and a history of prior severe COPD exacerbations requiring hospitalization have been shown to increase the risk of patients with COPD developing CAP [4].

Effective COPD pharmacotherapy is important to help improve patients’ quality of life and prevent exacerbations [5]. The Global Initiative for Chronic Obstructive Pulmonary Disease (GOLD) 2025 report recommends dual therapy (long-acting β2-agonist [LABA]/long-acting muscarinic antagonist [LAMA]) or triple therapy (inhaled corticosteroid [ICS]/LABA/LAMA) as preferred treatments for patients with COPD who are at risk of exacerbations in GOLD group E [6]. Single-inhaler triple therapy (SITT) has been shown to be more convenient and effective than multiple-inhaler triple therapy (MITT) in improving adherence [6].

The most recent Japanese Respiratory Society guidelines (6th edition) recommend the addition of an ICS for patients with COPD with concomitant asthmatic features and/or frequent exacerbations as well as blood eosinophilia [7]. Previous studies have reported that the use of an ICS in COPD treatment is related to the risk of pneumonia [8,9]. Therefore, the use of ICS-containing treatments for COPD should be carefully considered according to the individual patient’s condition.

Fluticasone furoate/umeclidinium/vilanterol (FF/UMEC/VI) 100 µg is a SITT that has been approved as a maintenance treatment for patients with COPD since March 2019 [10]. The IMPACT trial demonstrated a lower rate of moderate or severe exacerbations with FF/UMEC/VI vs. FF/VI or UMEC/VI and a lower rate of COPD-related hospitalization with FF/UMEC/VI vs. UMEC/VI [11]. The IMPACT trial also demonstrated higher rates of pneumonia among patients treated with FF/UMEC/VI compared with patients treated with UMEC/VI [11]. There was no significant difference in the risk of pneumonia between patients treated with FF/UMEC/VI or FF/VI [11]. A sub-group analysis of Japanese patients in the IMPACT study found that the incidence of pneumonia among UMEC/VI users in the Japanese cohort was the same as the overall population (5% in both) [12], whereas the incidence among users of ICS-containing treatments in the Japanese cohort (FF/UMEC/VI users: 18%, FF/VI users: 21%) was higher compared with the overall population (FF/UMEC/VI users: 8%, FF/VI users: 7%) [12]. However, a separate follow-up study of the IMPACT trial investigating the risk of exacerbation and pneumonia with FF/UMEC/VI vs. UMEC/VI or FF/VI found that FF/UMEC/VI reduced the risk of combined moderate or severe exacerbations or pneumonia vs. FF/VI and UMEC/VI [13].

To our knowledge, there are no studies directly comparing the risk or incidence of hospitalization due to CAP among patients with COPD treated with FF/UMEC/VI compared with patients treated with MITT. Due to the more convenient dosing strategy of SITT compared with MITT, prescriptions for SITT are expected to increase. As a result, the Japanese Pharmaceuticals and Medical Devices Agency (PMDA) requested a post-marketing database surveillance study to be conducted to compare the risk of pneumonia between SITT and MITT use in real-world clinical settings. Therefore, the aim of this study is to investigate the occurrence of hospitalization due to CAP among patients with COPD in Japan who received FF/UMEC/VI 100 µg or MITT. This post-marketing database surveillance study was conducted in compliance with Good Post-Marketing Study Practice (GPSP) ordinance in Japan.

## 2. Materials and Methods

### 2.1. Study Design

**Figure 1:**
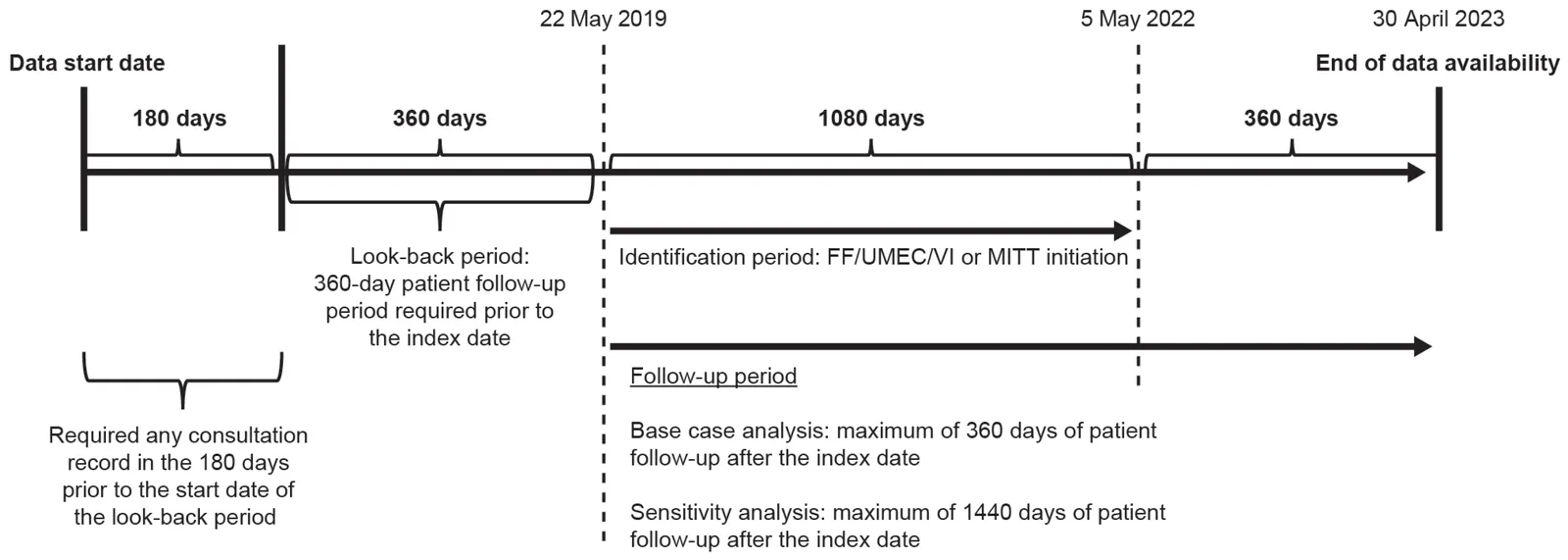
Study design. FF/UMEC/VI: fluticasone furoate/umeclidinium/vilanterol; MITT: multiple-inhaler triple therapy.

This was a retrospective cohort study using health insurance hospital claims data provided by the Medical Data Vision (MDV) Co., Ltd. (Tokyo, Japan) database. The patient identification period was from 22 May 2019 (the date when FF/UMEC/VI became available to use in Japan) to 5 May 2022 (Figure 1). The index date was defined as the first prescription of FF/UMEC/VI 100 µg or MITT. Patients were followed-up from the index date to hospitalization admission due to CAP, the end of the exposure period with FF/UMEC/VI or MITT due to discontinuation, 360 days after the index date, or patient death, whichever occurred first. The 360-day follow-up period was based on a 2015 study by Suissa et al. [14], which also used a 360-day follow-up period to assess the discontinuation of ICSs and risk reduction in pneumonia among patients with COPD.

Definitions for treatment continuation and discontinuation were based on prescription records using the concepts of the prescription period, prescription gaps, and the grace period. The prescription period was the period from the date of prescription until the end of treatment. Estimates were based on the dosage information provided in the package insert for each drug. The prescription gap was the number of days from the last day of the prescription period for the previous prescription to the start date of the next prescription. The grace period was defined as a 30-day allowable gap to determine whether treatment was continued or discontinued. Treatment was considered continued if the gap was 30 days and discontinued if the gap was ≥31 days. If the final prescription period started before the end date of the previous prescription period, the overlapping days of the two prescription periods were added to the grace period to calculate the end date of the final prescription period.

### 2.2. Data Source

This study was conducted using available data from the Japanese MDV hospital claims database from November 2017 through to April 2023. The MDV database covers approximately 25% of acute-care hospitals in Japan, includes both inpatient and outpatient services, and is updated monthly to reduce the delay for data access and analyses. The database also contains disease diagnoses, claims for medical procedures and pharmacy prescriptions, and laboratory test results, which are available for approximately 10% of patients. Each Diagnosis Procedure Combination (DPC) hospital included in the MDV database assigned a hospital-specific ID to each patient so they could be followed both as inpatients and outpatients in the same DPC hospital but could not be followed after a transfer.

### 2.3. Study Population

To be included in the study, patients must have been ≥40 years of age at the time of index and had at least one prescription of FF/UMEC/VI or MITT between 22 May 2019 and 5 May 2022. Patients were also required to have at least one inpatient or outpatient diagnosis of COPD (International Classification of Diseases 10th revision [ICD-10] code J42, J43, or J44) at the time of index and at least two diagnoses of COPD during the 360 days prior to the index date (look-back period), and at least one record with any ICD-10 code in the 180 days prior to the start date of the look-back period.

Patients were excluded if they had a hospitalization due to CAP in the 30 days before, but not including, the index date. Hospitalization due to CAP was defined using ICD-10 codes (J10.0, J11.0, or J12–18) for pneumonia, an ICD-10 code for pneumonia in DPC disease segment 21 (disease which triggered the hospitalization), a prescription of antibiotics (Anatomical Therapeutic Chemical code J01) on the day of hospital admission or the next day of hospitalization, and diagnostic imaging conducted between 2 days before and 7 days after the hospitalization.

Incident users were a subpopulation of overall users (patients who started FF/UMEC/VI or MITT between 22 May 2019 and 5 May 2022) who did not use any triple therapies in the 360 days prior to the index date. MITT users were defined as patients with overlapping prescriptions of ICS, LABA, and LAMA in either two or three devices of ≥1 day.

### 2.4. Study Objectives

The primary objective of this study was to compare the occurrence of hospitalization due to CAP among patients with COPD who were incident users of FF/UMEC/VI or MITT.

There were two secondary objectives of this study. The first was to estimate the incidence rate of hospitalization due to CAP among patients with COPD who were treated with FF/UMEC/VI or MITT (overall and incident users). The second was to describe the characteristics of patients with COPD who were treated with FF/UMEC/VI or MITT (overall and incident users).

### 2.5. Sensitivity Analyses

Three sensitivity analyses were performed to confirm the robustness of the analysis of the primary and secondary objectives. The first was when the eligibility criteria were changed so that a prescription of any inhaled medicine with an indication of COPD, at the index date and on at least two outpatient or hospital admission visits in the look-back period, was also required alongside a COPD diagnosis with an ICD-10 code for COPD (J42, J43, or J44). This was to increase the specificity of identifying COPD. As the definition of MITT exposure requiring only one day of overlap of ICS, LABA, and LAMA on the index date may overestimate MITT exposure, another sensitivity analysis was performed where the required duration of an overlap of three components for the MITT group was changed from ≥1 day to ≥14 days (including the index date). For the final sensitivity analysis the follow-up period was changed from a maximum of 360 days to a maximum of 1440 days (after the index date), for long-term results.

### 2.6. Data Analysis

Inverse probability of treatment weighting (IPTW) Cox models based on propensity scores (PS) [15] were used to estimate hazard ratios (HRs) and 95% confidence intervals (CIs) for hospitalization due to CAP. The PS was used to adjust for the following covariates, using logistic regression: sex gender, age, calendar year of index date, month of index date, COPD treatments in the look-back period, hospitalization due to COPD exacerbation in the look-back period, hospitalization due to CAP in the look-back period, comorbidities of pre-defined diseases (e.g., asthma, myocardial infarction, congestive heart failure, cerebrovascular disease, peptic ulcer, peripheral vascular disease, connective tissue disease, diabetes, anxiety, and depression), body mass index (BMI), smoking history, and execution of lung-function test. Multicollinearity was considered when selecting covariates. Variance estimation was calculated using the robust sandwich variance method [16].

Incidence rates for hospitalization due to CAP and 95% CIs of outcomes were estimated using a Poisson regression model and expressed per 1000 person-years.

IPTW using a PS model (which was calculated for each patient using logistic regression) was used to estimate descriptive statistics and standardized differences for the characteristics of unweighted and weighted incident FF/UMEC/VI and MITT users. Descriptive characteristics included mean and standard deviation (SD) for categorical variables and relative proportions for continuous variables. Standardized differences were used to assess differences in patient characteristics [17], with a standardized difference of <10% indicating non-significance.

For the time to the first occurrence of hospitalization due to CAP, the probability of an event in the cohorts was illustrated using a non-parametric cumulative incidence function for overall and incident users in the competing event setting (death was considered the competing event). The cumulative incidence of hospitalization due to CAP was also plotted using IPTW in incident users only.

### 2.7. Adjustments for Missing BMI Data

Of the covariates included in the model, BMI data were frequently missing. BMI has been shown to be a risk factor for ICS-related pneumonia [18] and the many missing data may not appropriately adjust the analysis. However, it was unclear whether missing BMI data were “missing completely at random”, “missing at random”, or “missing not at random.” The following four adjustments were made to address all missing patterns [19]: covariate adjustment including BMI without BMI missing imputation (complete case analysis); covariate adjustment without BMI; covariate adjustment with multiple imputation for BMI; and covariate adjustment with the missing-indicator method for BMI. The results obtained after the covariates were adjusted with multiple imputation and missing-indicator methods are presented in the Results section, and the results for the complete case analysis and for when covariates were adjusted without BMI are presented in the Supplementary Files.

### 2.8. Ethics Approval and Informed Consent

This study was reviewed and approved by the Ethics Review Board of Kitamachi Clinic (central ethics committee) (approval number: GSK08152). This study complied with all applicable laws regarding subject privacy, according to the Declaration of Helsinki. No direct subject contact or primary collection of individual human subject data have occurred in this study. This study used existing, fully de-identified data and the subject(s) cannot be identified, directly or through identifiers; therefore, informed consent was not required. Study results were in tabular form and aggregate analyses that omit subject identification. This study was also conducted in compliance with GPSP ordinance in Japan.

## 3. Results

### 3.1. Patient Attrition

**Figure 2:**
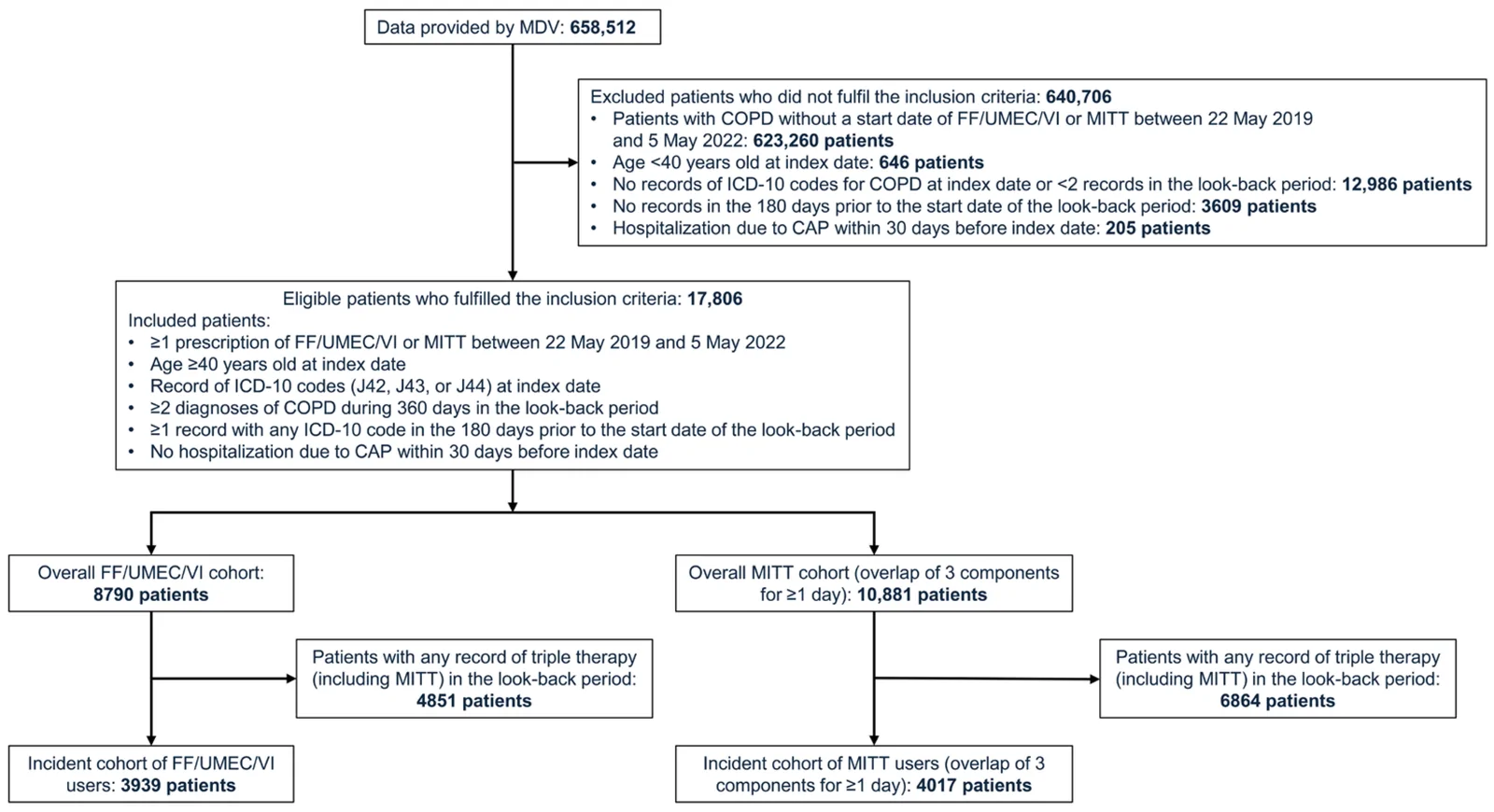
Patient attrition. CAP: community-acquired pneumonia; COPD: chronic obstructive pulmonary disease; FF/UMEC/VI: fluticasone furoate/umeclidinium/vilanterol; ICD-10: International Classification of Diseases 10th revision; MDV: Medical Data Vision; MITT: multiple-inhaler triple therapy.

In total, 8790 patients were included in the overall FF/UMEC/VI cohort and 10,881 patients were included in the overall MITT cohort (Figure 2). Of these patients, 3939 and 4017 were included in the incident FF/UMEC/VI and MITT cohorts, respectively.

### 3.2. Baseline Patient Sociodemographic and Clinical Characteristics

#### 3.2.1. Overall Users

The mean (SD) age was 74.2 (9.2) years and 73.1 (10.5) years in the FF/UMEC/VI and MITT cohorts, respectively (Table 1). The FF/UMEC/VI cohort had a higher proportion of male patients than the MITT cohort (79.3% vs. 69.7%). A lower proportion of patients had asthma in the FF/UMEC/VI cohort compared with the MITT cohort (56.7% vs. 85.7%). The proportion of patients with a recorded ICS prescription in the year prior to the index date was 67.1% in the FF/UMEC/VI cohort and 80.0% in the MITT cohort. The mean (SD) observation period was 222.5 (125.8) days and 159.7 (120.3) days in the FF/UMEC/VI and MITT cohorts, respectively. A total of 45.8% of patients in the FF/UMEC/VI cohort and 45.7% patients in the MITT cohort had missing BMI data. Mean (SD) BMI was 22.4 (4.1) and 22.5 (4.5) in the FF/UMEC/VI and MITT cohorts, respectively.

#### 3.2.2. Incident Users

The mean (SD) age was 74.9 (8.7) years and 73.3 (10.6) years in the FF/UMEC/VI and MITT cohorts, respectively (Table 1). The FF/UMEC/VI cohort had a higher proportion of male patients than the MITT cohort (81.3% vs. 69.4%). A lower proportion of patients had asthma in the FF/UMEC/VI cohort compared with the MITT cohort (31.1% vs. 76.4%). The proportion of patients with a recorded ICS prescription in the year prior to the index date was 27.6% in the FF/UMEC/VI cohort and 47.5% in the MITT cohort. The mean (SD) observation period was 208.0 (126.2) days and 126.9 (108.8) days in the FF/UMEC/VI and MITT cohorts, respectively. A total of 44.8% of patients in the FF/UMEC/VI cohort and 42.0% of patients in the MITT cohort had missing BMI data. Mean (SD) BMI was 22.3 (4.0) and 22.4 (4.4) in the FF/UMEC/VI and MITT cohorts, respectively.

The baseline demographics and clinical characteristics for patients when the PS was adjusted with multiple imputation for BMI are shown in Table S1. Baseline demographics and clinical characteristics for patients when the PS was adjusted with the missing-indicator method for BMI are shown in Table S2.

### 3.3. Hospitalization Due to CAP Among Incident Users of FF/UMEC/VI or MITT

The unadjusted HR (95% CI) for hospitalization due to CAP among incident users of FF/UMEC/VI compared with MITT when the PS was adjusted with multiple imputation for BMI and when the PS was adjusted with the missing indicator method for BMI was 0.92 (0.72–1.17). When the PS was adjusted with multiple imputation for BMI, the adjusted HR (95% CI) was 1.07 (0.78–1.47) (Table 2).

When the PS was adjusted with the missing-indicator method for BMI, the adjusted HR (95% CI) was 1.07 (0.78–1.48) (Table 2). The unadjusted and adjusted HRs (95% CIs) for hospitalization due to CAP among incident users of FF/UMEC/VI compared with MITT for the complete case analysis and for when the PS was adjusted without BMI are shown in Table S3.

The unadjusted and adjusted HRs (95% CIs) for hospitalization due to CAP among incident users of FF/UMEC/VI compared with MITT for the sensitivity analyses are shown in Tables S4–S6.

### 3.4. Incidence Rates of Hospitalization Due to CAP

#### 3.4.1. Overall Users

Among overall users of FF/UMEC/VI and MITT, the unadjusted incidence rate (95% CI) for hospitalization due to CAP when using either multiple imputation or the missing-indicator method for BMI was 60.01 (53.81–66.93) per 1000 patient-years in the FF/UMEC/VI cohort and 73.73 (66.40–81.88) per 1000 patient-years in the MITT cohort (Table 3).

The adjusted incidence rate (95% CI) when using multiple imputation for BMI was 226.62 (161.85–317.31) and 289.22 (204.64–408.75) per 1000 patient-years in the FF/UMEC/VI and MITT cohorts, respectively (Table 3).

The adjusted incidence rate (95% CI) when using the missing-indicator method for BMI was 219.91 (156.55–308.90) and 280.27 (197.23–398.27) per 1000 patient-years in the FF/UMEC/VI and MITT cohorts, respectively (Table 3).

The unadjusted and adjusted incidence rates (95% CIs) for hospitalization due to CAP among overall users of FF/UMEC/VI compared with MITT for the complete case analysis and the analysis where covariates were adjusted without BMI are shown in Table S7.

The unadjusted and adjusted incidence rates (95% CIs) for hospitalization due to CAP among overall users of FF/UMEC/VI compared with MITT for the sensitivity analyses are shown in Tables S8–S10.

#### 3.4.2. Incident Users

Among incident users of FF/UMEC/VI and MITT, the unadjusted incidence rate (95% CI) for hospitalization due to CAP when using either multiple imputation or the missing-indicator method for BMI was 63.29 (53.72–74.56) per 1000 patient-years in the FF/UMEC/VI cohort and 79.12 (65.61–95.41) per 1000 patient-years in the MITT cohort (Table 4).

The adjusted incidence rate (95% CI) when using multiple imputation for BMI was 160.95 (87.87–294.81) and 227.77 (122.05–425.05) per 1000 patient-years in the FF/UMEC/VI and MITT cohorts, respectively (Table 4).

The adjusted incidence rate (95% CI) when using the missing-indicator method for BMI was 158.53 (86.59–290.26) and 225.36 (119.90–423.61) per 1000 patient-years in the FF/UMEC/VI and MITT cohorts, respectively (Table 4).

The unadjusted and adjusted incidence rates (95% CIs) for hospitalization due to CAP among overall users of FF/UMEC/VI compared with MITT for the complete case analysis and the analysis where covariates were adjusted without BMI are shown in Table S11.

The unadjusted and adjusted incidence rates (95% CIs) for hospitalization due to CAP among overall users of FF/UMEC/VI compared with MITT for the sensitivity analyses are shown in Tables S12–S14.*

### 3.5. Time to Occurrence of Hospitalization Due to CAP

**Figure 3:**
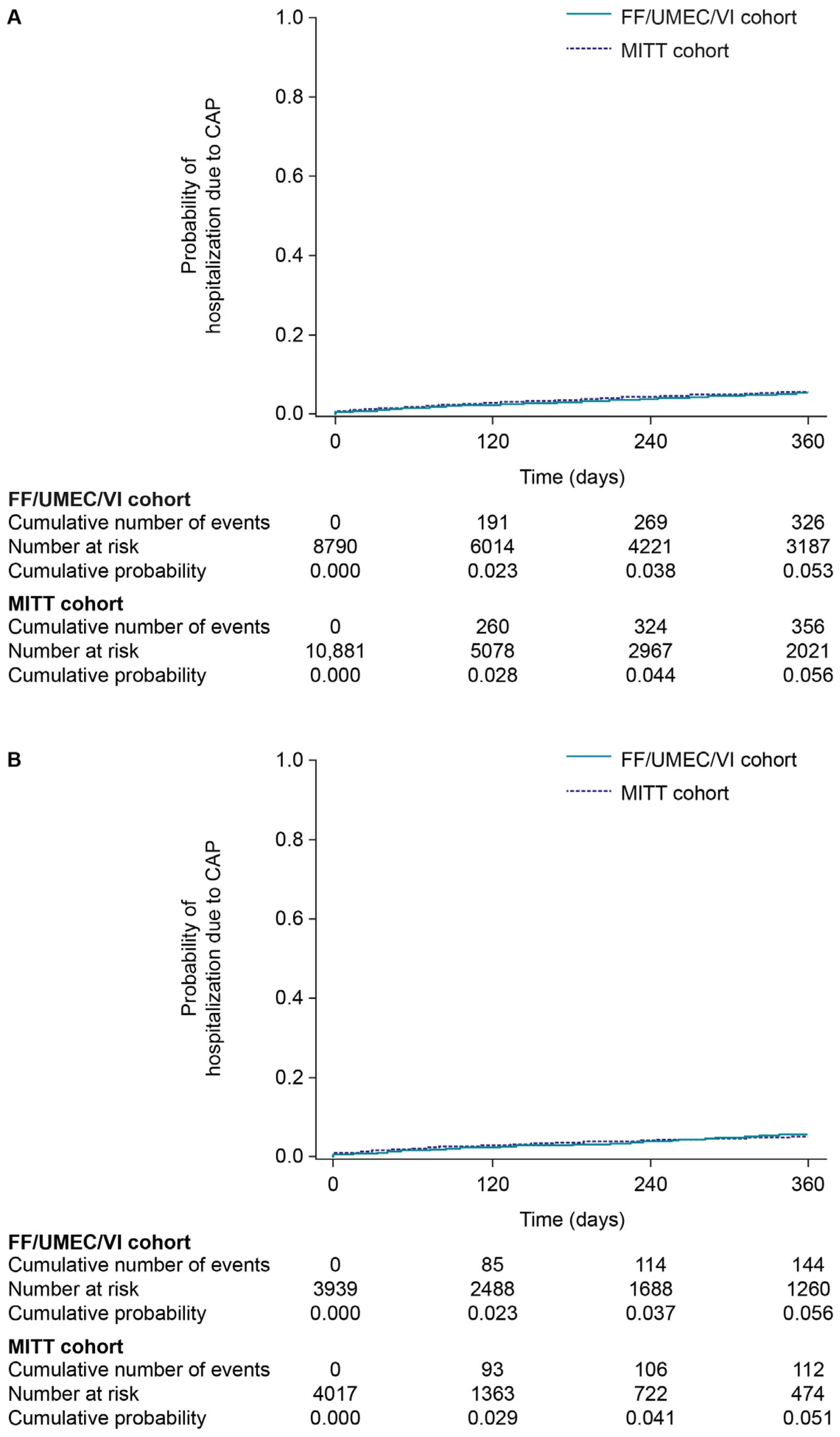
Cumulative unadjusted incidence of first occurrence of hospitalization due to CAP for (**A**) overall users of FF/UMEC/VI and MITT, and (**B**) incident users of FF/UMEC/VI and MITT. CAP: community-acquired pneumonia; FF/UMEC/VI: fluticasone furoate/umeclidinium/vilanterol; MITT: multiple-inhaler triple therapy.

The cumulative unadjusted incidence rate of first occurrence of hospitalization due to CAP at 360 days post-index was 0.053 and 0.056 in the FF/UMEC/VI and MITT cohorts, respectively, for overall users and 0.056 and 0.051 in the FF/UMEC/VI and MITT cohorts, respectively, for incident users (Figure 3A,B).

**Figure 4:**
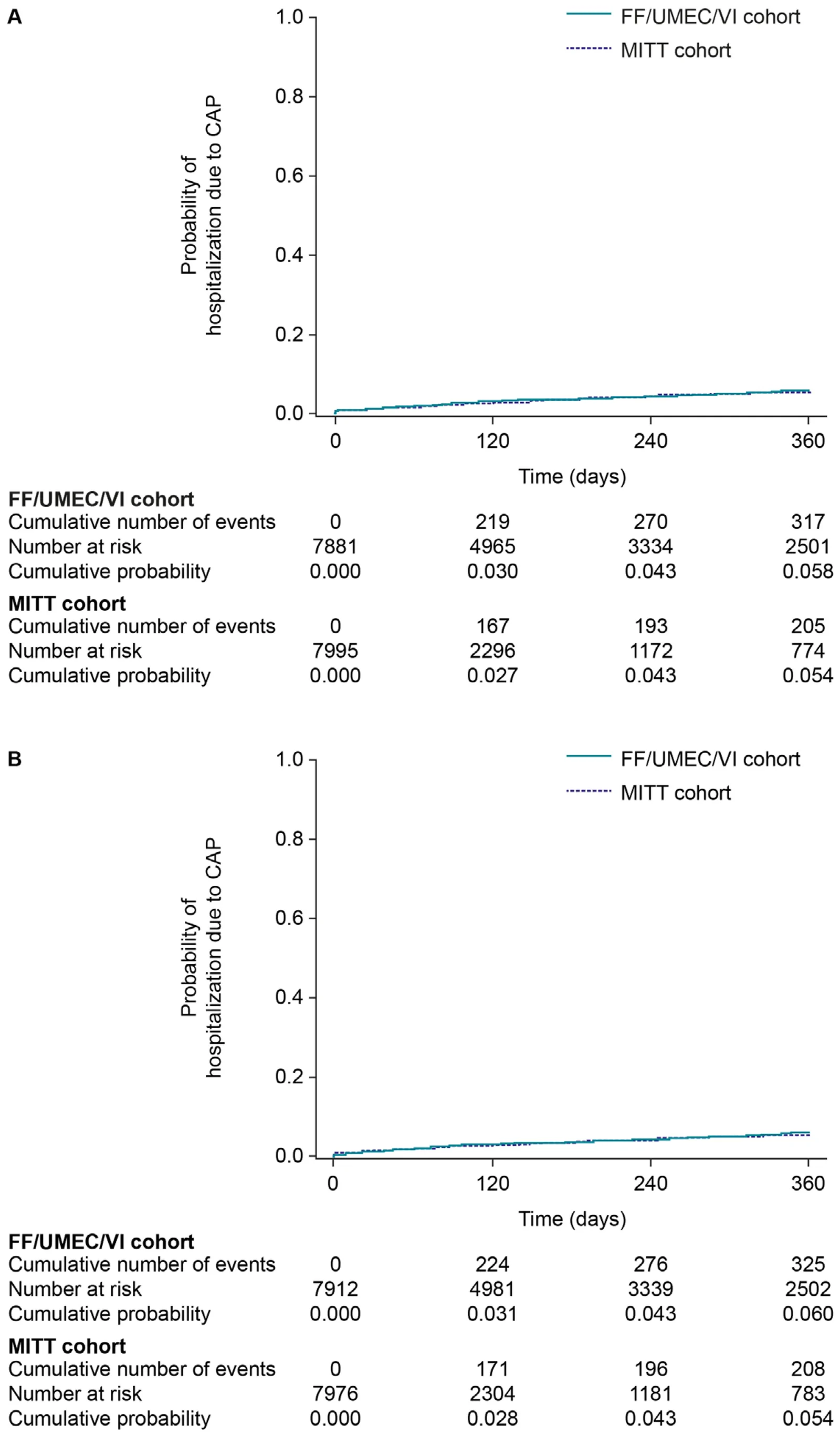
Cumulative incidence of first occurrence of hospitalization due to CAP for incident users of FF/UMEC/VI and MITT when PS is adjusted with (**A**) multiple imputation for BMI, and (**B**) missing-indicator method for BMI. BMI: body mass index; CAP: community-acquired pneumonia; FF/UMEC/VI: fluticasone furoate/umeclidinium/vilanterol; MITT: multiple-inhaler triple therapy; PS: propensity score.

When the PS was adjusted with multiple imputation for BMI, the cumulative incidence rate of first occurrence of hospitalization due to CAP at 360 days post-index among incident users of FF/UMEC/VI was 0.058 (Figure 4A). For the MITT cohort, the incidence rate was 0.054 (Figure 4A).

When the PS was adjusted with the missing-indicator method for BMI, the cumulative incidence rate of first occurrence of hospitalization due to CAP at 360 days post-index among incident users of FF/UMEC/VI was 0.060 (Figure 4B). For the MITT cohort, the incidence rate was 0.054 (Figure 4B).

The cumulative incidence rates for the complete case analysis and for when PS was adjusted without BMI are shown in Figure S1A,B.

## 4. Discussion

This post-marketing database surveillance study investigated the occurrence of hospitalization due to CAP among patients with COPD who were receiving FF/UMEC/VI 100 µg or MITT in a real-world setting in Japan.

The results for the primary objective of this study show that the risk of hospitalization due to CAP in the FF/UMEC/VI cohort was consistent across the different adjustment methods for BMI and the results of the sensitivity analyses confirmed the robustness of the results. Previous global studies have demonstrated similar risks of pneumonia in patients receiving SITT and MITT. The INTREPID trial demonstrated that the incidence of pneumonia-related serious adverse events was similar between patients receiving FF/UMEC/VI and non-ELLIPTA MITT [20]. A 2023 study that conducted a Delphi survey found that 81% of participants agreed that the risk of pneumonia was similar for SITTs and MITTs. [21]. This study also suggests that the risk of pneumonia would be similar in patients for all SITT combinations, due to pneumonia being an ICS-class effect [21].

In this current study, the incidence rate of hospitalization due to CAP in the FF/UMEC/VI cohort was numerically lower than that in the MITT cohort. A previous study investigating the risk of COPD exacerbation and pneumonia in patients with COPD found that 5%, 4%, and 3% of patients receiving FF/UMEC/VI, FF/VI, or UMEC/VI, respectively, had investigator-reported serious pneumonia resulting in hospitalization, prolonged hospitalization, or death [13]. However, the same study found that FF/UMEC/VI reduced the rate of investigator-reported or radiologically confirmed pneumonia resulting in hospitalization/death or a severe COPD exacerbation compared with FF/VI or UMEC/VI [13]. As the GOLD report recommends SITT over MITT due to the improved adherence that SITT offers [6], it is possible that patients had improved adherence to FF/UMEC/VI compared with MITT, which may have impacted the results, although medication adherence was not evaluated in this study.

Regarding the time to onset of hospitalization due to CAP between FF/UMEC/VI and MITT users, there were no significant differences between both cohorts. A previous study found that the HR (95% CI) for time to first hospitalization or death or severe exacerbation due to investigator-reported pneumonia was 0.92 (0.82–1.04) for FF/UMEC/VI vs. FF/VI and 0.87 (0.81–0.94) for FF/UMEC/VI vs. UMEC/VI [13].

### Limitations

This study used the MDV database, which consists of hospital data, and thus only patient data in the hospitals included in the MDV database are available. Patients who transferred to a hospital outside of the MDV database could not be followed up. Additionally, it is possible that some pneumonia events requiring hospitalization were recorded outside of DPCs, resulting in an underestimation of the outcome measure. If pneumonia requiring hospitalization was treated in hospitals outside of the MDV database, such data were not available, and the outcome measure would have been underestimated. Other limitations include those commonly associated with database studies, such as missing data (including BMI and smoking history, medical history or comorbidities in the look-back period, and pneumonia treatment in the look-back period at hospitals outside of the database) or incomplete patient records. Additionally, some key risk factors for pneumonia, such as lung function and respiratory symptoms, were not available in the MDV database. Despite adjusting for differences using IPTW based on propensity scores, there is a potential for residual confounding due to unobserved parameters that may impact prescribers’ treatment preferences. We anticipate that the above limitations affect both the FF/UMEC/VI and MITT cohorts to a similar extent and therefore any impact on the validity of the study would be minimal.

## 5. Conclusions

Our study showed no difference in the risk of CAP among patients with COPD in Japan who were treated with FF/UMEC/VI compared with MITT; however, results should be carefully interpreted based on the limitations. This study provides important safety information that may help healthcare providers select an appropriate treatment for patients with COPD in Japan.

## Figures and Tables

**Table 1:** Baseline demographics and clinical characteristics.

	Overall Users			Incident Users		
Characteristics	FF/UMEC/VI Cohort (*n* = 8790)	MITT Cohort (*n* = 10,881)	*p*-Value ^a^	FF/UMEC/VI Cohort (*n* = 3939)	MITT Cohort (*n* = 4017)	*p*-Value ^a^
Sex, *n* (%)						
Male	6971 (79.3)	7588 (69.7)	*p* < 0.001 ***	3204 (81.3)	2788 (69.4)	*p* < 0.001 ***
Female	1819 (20.7)	3293 (30.3)		735 (18.7)	1229 (30.6)	
Age at index date, years						
Mean (SD)	74.2 (9.2)	73.1 (10.5)	*p* < 0.001 ***	74.9 (8.7)	73.3 (10. 6)	*p* < 0.001 ***
40 to <65, *n* (%)	1172 (13.3)	1957 (18.0)	*p* < 0.001 ***	416 (10.6)	694 (17.3)	*p* < 0.001 ***
65≤ to <75, *n* (%)	2940 (33.4)	3431 (31.5)		1305 (33.1)	1212 (30.2)	
75≤ to <85, *n* (%)	3692 (42.0)	4239 (39.0)		1741 (44.2)	1642 (40.9)	
≥85, *n* (%)	986 (11.2)	1254 (11.5)		477 (12.1)	469 (11.7)	
Calendar year of index, ^b^ *n* (%)						
2019	2558 (29.1)	4520 (41.5)	*p <* 0.001 ***	744 (18.9)	1266 (31.5)	*p* < 0.001 ***
2020	2858 (32.5)	3656 (33.6)		1432 (36.4)	1493 (37.2)	
2021	2632 (29.9)	2187 (20.1)		1385 (35.2)	1008 (25.1)	
2022	742 (8.4)	518 (4.8)		378 (9.6)	250 (6.2)	
Month of index date, *n* (%)						
January	718 (8.2)	848 (7.8)	*p <* 0.001 ***	352 (8.9)	361 (9.0)	*p* < 0.001 ***
February	647 (7.4)	648 (6.0)		314 (8.0)	280 (7.0)	
March	740 (8.4)	633 (5.8)		390 (9.9)	271 (6.7)	
April	684 (7.8)	654 (6.0)		350 (8.9)	277 (6.9)	
May	386 (4.4)	809 (7.4)		186 (4.7)	275 (6.8)	
June	578 (6.6)	1333 (12.3)		246 (6.2)	423 (10.5)	
July	839 (9.5)	1248 (11.5)		312 (7.9)	376 (9.4)	
August	849 (9.7)	1045 (9.6)		294 (7.5)	333 (8.3)	
September	810 (9.2)	967 (8.9)		331 (8.4)	333 (8.3)	
October	836 (9.5)	931 (8.6)		371 (9.4)	360 (9.0)	
November	828 (9.4)	858 (7.9)		362 (9.2)	344 (8.6)	
December	875 (10.0)	907 (8.3)		431 (10.9)	384 (9.6)	
COPD treatments in the look-back period, ^c^ *n* (%)						
LAMA	2597 (29.5)	4359 (40.1)	*p* < 0.001 ***	281 (7.1)	634 (15.8)	*p* < 0.001 ***
LABA	89 (1.0)	71 (0.7)	*p* = 0.006 **	78 (2.0)	51 (1.3)	*p* = 0.013 *
LABA/LAMA	2447 (27.8)	1734 (15.9)	*p* < 0.001 ***	1605 (40.7)	957 (23.8)	*p* < 0.001 ***
ICS/LABA	2050 (23.3)	3047 (28.0)	*p* < 0.001 ***	547 (13.9)	759 (18.9)	*p* < 0.001 ***
ICS/LABA/LAMA	1124 (12.8)	632 (5.8)	*p* < 0.001 ***	0 (0)	0 (0)	–
Maintenance therapy status						
No maintenance therapy	1876 (21.3)	2762 (25.4)	*p* < 0.001 ***	1447 (36.7)	1642 (40.9)	*p* < 0.001 ***
LABA or LAMA monotherapy	1311 (14.9)	2693 (24.7)		318 (8.1)	620 (15.4)	
LABA/LAMA or ICS/LABA dual therapy	2558 (29.1)	2730 (25.1)		2174 (55.2)	1755 (43.7)	
ICS/LABA/LAMA triple therapy	3045 (34.6)	2696 (24.8)		0 (0)	0 (0)	
ICS	5897 (67.1)	8707 (80.0)	*p* < 0.001 ***	1089 (27.6)	1910 (47.5)	*p* < 0.001 ***
OCS	1993 (22.7)	3304 (30.4)	*p* < 0.001 ***	718 (18.2)	1110 (27.6)	*p* < 0.001 ***
Home oxygen therapy	1077 (12.3)	1375 (12.6)	*p* = 0.422	357 (9.1)	304 (7.6)	*p* = 0.017 *
Hospitalization due to COPD exacerbation in the look-back period, ^d^ *n* (%)	573 (6.5)	864 (7.9)	*p* < 0.001 ***	216 (5.5)	305 (7.6)	*p* < 0.001 ***
Hospitalization due to CAP in the look-back period, ^d^ *n* (%)	504 (5.7)	759 (7.0)	*p* < 0.001 ***	163 (4.1)	195 (4.9)	*p* = 0.130
Comorbidities, ^d^ *n* (%)						
Asthma (ICD-10 codes only)	6420 (73.0)	9522 (87.5)	*p* < 0.001 ***	2242 (56.9)	3219 (80.1)	*p* < 0.001 ***
Asthma (ICD-10 codes and prescription data)	4982 (56.7)	9330 (85.7)	*p* < 0.001 ***	1226 (31.1)	3068 (76.4)	*p* < 0.001 ***
Myocardial infarction	452 (5.1)	543 (5.0)	*p* = 0.647	216 (5.5)	213 (5.3)	*p* = 0.728
Congestive heart failure	2622 (29.8)	3471 (31.9)	*p* = 0.002 **	1236 (31.4)	1326 (33.0)	*p* = 0.125
Cerebrovascular disease	1301 (14.8)	1601 (14.7)	*p* = 0.872	597 (15.2)	642 (16.0)	*p* = 0.322
Dementia	0 (0)	1 (0.0)	*p* = 1.000	0 (0)	1 (0.0)	*p* = 1.000
Peptic ulcer	2326 (26.5)	3060 (28.1)	*p* = 0.010 **	1046 (26.6)	1107 (27.6)	*p* = 0.325
Peripheral vascular disease	1122 (12.8)	1296 (11.9)	*p* = 0.070	571 (14.5)	541 (13.5)	*p* = 0.196
Connective tissue disease	666 (7.6)	872 (8.0)	*p* = 0.262	287 (7.3)	352 (8.8)	*p* = 0.017 *
Diabetes	3111 (35.4)	3910 (35.9)	*p* = 0.436	1394 (35.4)	1457 (36.3)	*p* = 0.413
Anxiety	472 (5.4)	701 (6.4)	*p* = 0.002 **	206 (5.2)	260 (6.5)	*p* = 0.019 *
Depression	447 (5.1)	707 (6.5)	*p* < 0.001 ***	212 (5.4)	271 (6.7)	*p* = 0.011 *
BMI ^d,e^						
Mean (SD)	22.42 (4.14)	22.52 (4.52)	*p* = 0.246	22.33 (4.03)	22.41 (4.39)	*p* = 0.522
<18.5, *n* (%)	821 (9.3)	1089 (10.0)	*p* = 0.006 **	362 (9.2)	423 (10.5)	*p* = 0.135
≥18.5 to <25, *n* (%)	2813 (32.0)	3307 (30.4)		1322 (33.6)	1348 (33.6)	
≥25, *n* (%)	1126 (12.8)	1508 (13.9)		492 (12.5)	559 (13.9)	
Missing data, *n* (%)	4030 (45.8)	4977 (45.7)		1763 (44.8)	1687 (42.0)	
Smoking history, ^d,e^ *n* (%)						
No data	5694 (64.8)	7423 (68.2)	*p* < 0.001 ***	2520 (64.0)	2656 (66.1)	*p* = 0.046 *
Smoker	3096 (35.2)	3458 (31.8)		1419 (36.0)	1361 (33.9)	
Lung-function test, ^d^ *n* (%)	3722 (42.3)	3922 (36.0)	*p* < 0.001 ***	1603 (40.7)	1516 (37.7)	*p* = 0.007 **
Mean (SD) follow-up period, days	222.5 (125.76)	159.7 (120.33)	*p* < 0.001 ***	208.0 (126.20)	126.9 (108.84)	*p* < 0.001 ***

No *p*-value = ≥ 0.05. * *p* < 0.05. ** *p* < 0.01. *** *p* < 0.001. ^a^ Regarding the test for differences between the FF/UMEC/VI cohort and the MITT cohort of patient information, Fisher’s exact test, χ^2^ test, or analysis of variance analysis was performed. ^b^ Index date is identified between 22 May 2019 and 5 May 2022. ^c^ LAMA, LABA, LABA/LAMA, and ICS/LABA treatments are identified during 180 days before the index date. ^d^ Assessment period is identified during 360 days before the index date. ^e^ Data were available from the hospitalization record. BMI: body mass index; CAP: community-acquired pneumonia; COPD: chronic obstructive pulmonary disease; FF/UMEC/VI: fluticasone furoate/umeclidinium/vilanterol; ICD-10: International Classification of Diseases 10th revision; ICS: inhaled corticosteroid; LABA: long-acting β2-agonist; LAMA: long-acting muscarinic antagonist; MITT: multiple-inhaler triple therapy; OCS: oral corticosteroid; SD: standard deviation.

**Table 2:** Hospitalization due to CAP among incident users of FF/UMEC/VI or MITT.

	PS Adjustment with Multiple Imputation for BMI		PS Adjustment with Missing-Indicator Method for BMI	
	FF/UMEC/VI Incident Users (*n* = 3939)	MITT Incident Users (*n* = 4017)	FF/UMEC/VI Incident Users (*n* = 3939)	MITT Incident Users (*n* = 4017)
Total patient-years	2275	1416	2275	1416
Events	144	112	144	112
FF/UMEC/VI vs. MITT unadjusted HR (95% CI)	0.92 (0.72–1.17)		0.92 (0.72–1.17)	
FF/UMEC/VI vs. MITT adjusted HR (95% CI)	1.07 (0.78–1.47)		1.07 (0.78–1.48)	

BMI: body mass index; CAP: community-acquired pneumonia; CI: confidence interval; FF/UMEC/VI: fluticasone furoate/umeclidinium/vilanterol; HR: hazard ratio; MITT: multiple-inhaler triple therapy; PS: propensity score.

**Table 3:** Incidence rates of hospitalization due to CAP—overall users.

	Covariate Adjustment with Multiple Imputation for BMI		Covariate Adjustment with Missing-Indicator Method for BMI	
	FF/UMEC/VI Overall Users (*n* = 8790)	MITT Overall Users (*n* = 10,881)	FF/UMEC/VI Overall Users (*n* = 8790)	MITT Overall Users (*n* = 10,881)
Total patient-years	5432	4828	5432	4828
Events	326	356	326	356
Unadjusted incidence rate (95% CI) per 1000 patient-years	60.01 (53.81–66.93)	73.73 (66.40–81.88)	60.01 (53.81–66.93)	73.73 (66.40–81.88)
Adjusted incidence rate (95% CI) per 1000 patient-years	226.62 (161.85–317.31)	289.22 (204.64–408.75)	219.91 (156.55–308.90)	280.27 (197.23–398.27)

BMI: body mass index; CAP: community-acquired pneumonia; CI: confidence interval; FF/UMEC/VI: fluticasone furoate/umeclidinium/vilanterol; MITT: multiple-inhaler triple therapy.

**Table 4:** Incidence rates of hospitalization due to CAP—incident users.

	Covariate Adjustment with Multiple Imputation for BMI		Covariate Adjustment with Missing-Indicator Method for BMI	
	FF/UMEC/VI Incident Users (*n* = 3939)	MITT Incident Users (*n* = 4017)	FF/UMEC/VI Incident Users (*n* = 3939)	MITT Incident Users (*n* = 4017)
Total patient-years	2275	1416	2275	1416
Events	144	112	144	112
Unadjusted incidence rate (95% CI) per 1000 patient-years	63.29 (53.72–74.56)	79.12 (65.61–95.41)	63.29 (53.72–74.56)	79.12 (65.61–95.41)
Adjusted incidence rate (95% CI) per 1000 patient-years	160.95 (87.87–294.81)	227.77 (122.05–425.05)	158.53 (86.59–290.26)	225.36 (119.90–423.61)

BMI: body mass index; CAP: community-acquired pneumonia; CI: confidence interval; FF/UMEC/VI: fluticasone furoate/umeclidinium/vilanterol; MITT: multiple-inhaler triple therapy.

## Data Availability

The data analyzed in this publication are derived from the MDV database (Tokyo, Japan). Authors had access to the study data for the purposes of this work only. The interpretation and conclusions contained in this study are those of the authors alone. Data were accessed through an existing GSK license to address the prespecified research questions only; therefore, the data cannot be broadly disclosed or made publicly available at this time. Access to the database can be requested via the website.

## Supplementary Materials

The following supporting information can be downloaded at: https://www.mdpi.com/article/10.3390/jcm14134697/s1, Table S1. Baseline demographics and clinical characteristics—PS adjustment with multiple imputation for BMI; Table S2. Baseline demographics and clinical characteristics—PS adjustment with missing-indicator methods for BMI; Table S3. Hospitalization due to CAP among incident users of FF/UMEC/VI or MITT—complete case analysis and PS adjustment without BMI; Table S4. Hospitalization due to CAP among incident users of FF/UMEC/VI or MITT—sensitivity analysis (if COPD is defined on the condition that the patient has a record of prescription of an inhaled COPD medicine in addition to the diagnosis); Table S5. Hospitalization due to CAP among incident users of FF/UMEC/VI or MITT—sensitivity analysis (if the definition of MITT is based on at least 14 days of overlapping prescription periods for three components); Table S6. Hospitalization due to CAP among incident users of FF/UMEC/VI or MITT—sensitivity analysis (when the maximum observation period was 1440 days after the index date); Table S7. Incidence rates of hospitalization due to CAP among overall users of FF/UMEC/VI or MITT—complete case analysis and covariate adjustment without BMI; Table S8. Incidence rates of hospitalization due to CAP among overall users of FF/UMEC/VI or MITT—sensitivity analysis (if COPD is defined on the condition that the patient has a record of prescription of an inhaled COPD medicine in addition to the diagnosis); Table S9. Incidence rates of hospitalization due to CAP among overall users of FF/UMEC/VI or MITT—sensitivity analysis (if the definition of MITT is based on at least 14 days of overlapping prescription periods for three components); Table S10. Incidence rates of hospitalization due to CAP among overall users of FF/UMEC/VI or MITT—sensitivity analysis (when the maximum observation period was 1440 days after the index date); Table S11. Incidence rates of hospitalization due to CAP among incident users of FF/UMEC/VI or MITT—complete case analysis and covariate adjustment without BMI; Table S12. Incidence rates of hospitalization due to CAP among incident users of FF/UMEC/VI or MITT—sensitivity analysis (if COPD is defined on the condition that the patient has a record of prescription of an inhaled COPD medicine in addition to the diagnosis); Table S13. Incidence rates of hospitalization due to CAP among incident users of FF/UMEC/VI or MITT—sensitivity analysis (if the definition of MITT is based on at least 14 days of overlapping prescription periods for three components); Table S14. Incidence rates of hospitalization due to CAP among incident users of FF/UMEC/VI or MITT—sensitivity analysis (when the maximum observation period was 1440 days after the index date); Figure S1. Cumulative incidence of first occurrence of hospitalization due to CAP for incident FF/UMEC/VI and MITT users for (A) the complete case analysis, and (B) when PS is adjusted without BMI.
