# Correction: Akiyama et al. Comparison of the Risk of Pneumonia Between Fluticasone Furoate/Umeclidinium/Vilanterol and Multiple-Inhaler Triple Therapy in Patients with COPD Using Health Insurance Claims Data: Final Analysis of Post-Marketing Database Surveillance in Japan. *J. Clin. Med.* 2025, *14*, 4697

**DOI:** 10.3390/jcm15135093

**Published:** 2026-06-30

**Authors:** Shoko Akiyama, Kenji Oda, Hiroko Mizohata, Natsuki Sasakura, Kenichi Hashimoto, Hiroki Maruoka

**Affiliations:** 1 Real World Data Analytics, Japan Development, GSK, Tokyo 107-0052, Japan; shoko.2.akiyama@gsk.com (S.A.); kenji_oda1987@yahoo.co.jp (K.O.); 2 Global Real-World Evidence & Health Outcomes Research Japan, GSK, Tokyo 107-0052, Japan; hiroko.2.mizohata@gsk.com (H.M.); kenichi.2.hashimoto@gsk.com (K.H.); 3 Respiratory Medical Affairs, Japan Medical Affairs, GSK, Tokyo 107-0052, Japan; natsuki.x.sasakura@gsk.com

## Text Correction

There was an error in the original publication [1]. The term ‘PS adjustment’ or ‘PS- adjusted’ was used incorrectly to describe the unadjusted and adjusted incidence rate estimations for hospitalization due to community-acquired pneumonia. Propensity scores were not used; the adjusted incidence rates were estimated using multivariable Poisson regression models with covariates directly entered, after handling missing body mass index values using multiple imputation or the missing-indicator method. PS adjustment was applied correctly to the HR estimation in Section *3.3. Hospitalization Due to CAP Among Incident Users of FF/UMEC/VI or MITT* and to the cumulative curve in Section *3.5. Time to Occurrence of Hospitalization Due to CAP*. 

A correction has been made to Section *2.7. Adjustments for Missing BMI Data* and Section *3.4. Incidence Rates of Hospitalization Due to CAP* (Section 3.4.1. Overall Users and Section 3.4.2. Incident Users).

### 2.7. Adjustments for Missing BMI Data

Of the covariates included in the model, BMI data were frequently missing. BMI has been shown to be a risk factor for ICS-related pneumonia [18] and the many missing data may not appropriately adjust the analysis. However, it was unclear whether missing BMI data were “missing completely at random”, “missing at random”, or “missing not at random.” The following four adjustments were made to address all missing patterns [19]: covariate adjustment including BMI without BMI missing imputation (complete case analysis); covariate adjustment without BMI; covariate adjustment with multiple imputation for BMI; and covariate adjustment with the missing-indicator method for BMI. The results obtained after the covariates were adjusted with multiple imputation and missing-indicator methods are presented in the Results section, and the results for the complete case analysis and for when covariates were adjusted without BMI are presented in the Supplementary Files.

#### 3.4.1. Overall Users

Among overall users of FF/UMEC/VI and MITT, the unadjusted incidence rate (95% CI) for hospitalization due to CAP when using either multiple imputation or the missing-indicator method for BMI was 60.01 (53.81–66.93) per 1000 patient-years in the FF/UMEC/VI cohort and 73.73 (66.40–81.88) per 1000 patient-years in the MITT cohort (Table 3).

The adjusted incidence rate (95% CI) when using multiple imputation for BMI was 226.62 (161.85–317.31) and 289.22 (204.64–408.75) per 1000 patient-years in the FF/UMEC/VI and MITT cohorts, respectively (Table 3).

The adjusted incidence rate (95% CI) when using the missing-indicator method for BMI was 219.91 (156.55–308.90) and 280.27 (197.23–398.27) per 1000 patient-years in the FF/UMEC/VI and MITT cohorts, respectively (Table 3).

The unadjusted and adjusted incidence rates (95% CIs) for hospitalization due to CAP among overall users of FF/UMEC/VI compared with MITT for the complete case analysis and the analysis where covariates were adjusted without BMI are shown in Table S7.

The unadjusted and adjusted incidence rates (95% CIs) for hospitalization due to CAP among overall users of FF/UMEC/VI compared with MITT for the sensitivity analyses are shown in Tables S8–S10.

#### 3.4.2. Incident Users

Among incident users of FF/UMEC/VI and MITT, the unadjusted incidence rate (95% CI) for hospitalization due to CAP when using either multiple imputation or the missing-indicator method for BMI was 63.29 (53.72–74.56) per 1000 patient-years in the FF/UMEC/VI cohort and 79.12 (65.61–95.41) per 1000 patient-years in the MITT cohort (Table 4).

The adjusted incidence rate (95% CI) when using multiple imputation for BMI was 160.95 (87.87–294.81) and 227.77 (122.05–425.05) per 1000 patient-years in the FF/UMEC/VI and MITT cohorts, respectively (Table 4).

The adjusted incidence rate (95% CI) when using the missing-indicator method for BMI was 158.53 (86.59–290.26) and 225.36 (119.90–423.61) per 1000 patient-years in the FF/UMEC/VI and MITT cohorts, respectively (Table 4).

The unadjusted and adjusted incidence rates (95% CIs) for hospitalization due to CAP among overall users of FF/UMEC/VI compared with MITT for the complete case analysis and the analysis where covariates were adjusted without BMI are shown in Table S11.

The unadjusted and adjusted incidence rates (95% CIs) for hospitalization due to CAP among overall users of FF/UMEC/VI compared with MITT for the sensitivity analyses are shown in Tables S12–S14.*

Corrections have also been made to the Abstract, Tables 3 and 4, and Tables S7–S14, where PS has either been removed or amended to ‘covariate’.

The authors would like to emphasize Section *2.6. Data Analysis* correctly describes the statistical methodology. 

The authors state that the scientific conclusions are unaffected. This correction was approved by the Academic Editor. The original publication has also been updated. 
