# Supplementary material for: Comparison of the Risk of Pneumonia Between Fluticasone Furoate/Umeclidinium/Vilanterol and Multiple-Inhaler Triple Therapy in Patients with COPD Using Health Insurance Claims Data: Final Analysis of Post-Marketing Database Surveillance in Japan
Source: J Clin Med. 2025 Jul 2;14(13):4697. doi: 10.3390/jcm14134697 (PMC12251023; doi:10.3390/jcm14134697)
Supplement: Supplementary file 1 [file jcm-14-04697-s001.zip › jcm-3715993-supplementary.pdf]

Supplementary Materials

Furoate/Umeclidinium/Vilanterol and Multiple-Inhaler Triple Therapy in Patients with COPD Using Health Insurance Claims Data: Final Analysis of Post-Marketing Database Surveillance in Japan

Authors:

Shoko Akiyama [1], Kenji Oda [1,†], Hiroko Mizohata [2], Natsuki Sasakura [3], Kenichi Hashimoto [2] and Hiroki Maruoka [2,*]

Affiliations:

[1]Real World Data Analytics, Japan Development, GSK, Tokyo 107-0052, Japan; [2]Global Real-World Evidence & Health Outcomes Research Japan, GSK, Tokyo 107-0052, Japan; [3]Respiratory Medical Affairs, Japan Medical Affairs, GSK, Tokyo 107-0052, Japan

* Correspondence: hiroki.x.maruoka@gsk.com; Tel.: +81-80-2052-1044

† Affiliation at the time of study.

Table S1. Baseline demographics and clinical characteristics — PS adjustment with multiple imputation for BMI.

	Unweighted			Weighted		
**Characteristics**	**FF/UMEC/VI Incident Users (*n* = 3939)**	**MITT Incident Users (*n* = 4017)**	**Absolute Standardized Difference (%)**	**FF/UMEC/VI Incident Users [a] (*n* = 3939)**	**MITT Incident Users [a] (*n* = 4017)**	**Absolute Standardized Difference (%)**
Sex, %						
Male	81.3	69.4	28.0	76.0	75.3	1.7
Female	18.7	30.6		24.0	24.7	
Age at index date, years						
Mean (SD)	74.9 (8.7)	73.3 (10. 6)	–	74.6 (–)	74.4 (–)	–
40 to <65, %	10.6	17.3	19.5	13.4	13.7	1.0
65≤ to <75, %	33.1	30.2	6.4	30.6	30.0	1.2
75≤ to <85, %	44.2	40.9	6.7	43.1	43.5	0.8
≥85, %	12.1	11.7	1.3	12.9	12.7	0.6
Calendar year of index date, [b] %						
2019	18.9	31.5	29.4	25.3	25.3	0.1
2020	36.4	37.2	1.7	36.5	36.5	0.1
2021	35.2	25.1	22.1	30.2	30.0	0.3
2022	9.6	6.2	12.5	8.0	8.1	0.3
Month of index date, %						
January	8.9	9.0	0.2	9.0	8.7	1.1
February	8.0	7.0	3.8	7.1	7.1	0.3
March	9.9	6.7	11.4	8.5	8.4	0.2
April	8.9	6.9	7.4	8.1	8.5	1.3
May	4.7	6.8	9.1	5.4	5.6	0.9
June	6.2	10.5	15.5	7.4	8.1	2.3
July	7.9	9.4	5.1	9.1	8.9	0.5
August	7.5	8.3	3.1	8.5	8.1	1.6
September	8.4	8.3	0.4	8.5	8.0	1.8
October	9.4	9.0	1.6	9.0	9.4	1.3
November	9.2	8.6	2.2	8.8	8.6	0.8

December	10.9	9.6	4.6	10.6	10.6	0.2
COPD treatments in the look-back period, [c] %						
LAMA	7.1	15.8	27.4	11.4	11.5	0.5
LABA	2.0	1.3	5.6	1.6	1.3	2.3
LABA/LAMA	40.7	23.8	36.8	29.6	26.6	6.5
ICS/LABA	13.9	18.9	13.6	18.5	21.2	7.4
ICS/LABA/LAMA	0	0	–	0	0	––
Maintenance therapy status						
No maintenance therapy	36.7	40.9	8.5	39.5	39.8	0.8
LABA or LAMA monotherapy	8.1	15.4	23.0	11.7	11.6	0.3
LABA/LAMA or ICS/LABA dual therapy	55.2	43.7	23.2	48.8	48.6	0.6
ICS/LABA/LAMA triple therapy	0.0	0.0	–	0.0	0.0	–
ICS	27.6	47.5	42.0	41.1	44.1	6.2
OCS	18.2	27.6	22.5	23.2	23.2	0.1
Home oxygen therapy	9.1	7.6	5.4	8.3	8.3	0.2
Hospitalization due to COPD exacerbation in the look-back period, [d] %	5.5	7.6	8.5	7.4	6.9	1.7
Hospitalization due to CAP in the look-back period, [d] %	4.1	4.9	3.5	5.1	4.9	0.9
Comorbidities, [d] %						
Asthma (ICD-10 codes only)	56.9	80.1	–	71.2	60.1	–
Asthma (ICD-10 codes and prescription data)	31.1	76.4	101.8	53.6	53.7	0.3

Myocardial infarction	5.5	5.3	0.8	5.5	6.0	2.4
Congestive heart failure	31.4	33.0	3.5	32.7	33.0	0.6
Cerebrovascular disease	15.2	16.0	2.3	15.9	15.8	0.3
Dementia	0	0	–	0	0	–
Peptic ulcer	26.6	27.6	2.3	26.9	27.5	1.3
Peripheral vascular disease	14.5	13.5	3.0	14.4	14.5	0.3
Connective tissue disease	7.3	8.8	5.4	7.7	8.2	1.8
Diabetes	35.4	36.3	1.8	35.8	36.7	1.8
Anxiety	5.2	6.5	5.3	5.9	6.4	2.3
Depression	5.4	6.7	5.7	5.9	6.2	1.2
BMI [d,e]						
Mean (SD)	–	–	–	–	–	–
<18.5, %	16.4	17.3	2.3	17.0	17.1	0.2
≥18.5 to <25, %	61.1	57.5	7.4	59.4	59.0	0.7
≥25, %	22.4	25.2	6.5	23.6	23.9	0.7
Smoking history, [d,e] %						
No data	64.0	66.1	4.5	64.3	64.3	0
Smoker	36.0	33.9		35.7	35.7	
Lung-function test, [d] %	40.7	37.7	6.1	38.4	39.0	1.1

[a] Weighted mean, SD and proportion were calculated using inverse probability of treatment weighting. [b] Index date is identified between 22 May 2019 and 5 May 2022. [c] LAMA, LABA, LABA/LAMA, and ICS/LABA treatments are identified during 180 days before the index date. [d] Assessment period is identified during 360 days before the index date. [e] Data were available from the hospitalization record.

BMI: body mass index; CAP: community-acquired pneumonia; COPD: chronic obstructive pulmonary disease; FF/UMEC/VI: fluticasone furoate/umeclidinium/vilanterol; ICD-10: International Classification of Diseases 10th revision; ICS: inhaled corticosteroid; LABA: long-acting β2-agonist; LAMA: long-acting muscarinic antagonist; MITT: multiple-inhaler triple therapy; OCS: oral corticosteroid; PS: propensity score; SD: standard deviation.

Table S2. Baseline demographics and clinical characteristics — PS adjustment with missing-indicator methods for BMI.

	Unweighted			Weighted		
Characteristics	**FF/UMEC/VI Incident Users (*n* = 3939)**	**MITT Incident Users (*n* = 4017)**	**Absolute Standardized Difference (%)**	**FF/UMEC/VI Incident Users [a] (*n* = 3939)**	**MITT Incident Users [a] (*n* = 4017)**	**Absolute Standardized Difference (%)**
Sex, %						
Male	81.3	69.4	28.0	76.0	75.3	1.7
Female	18.7	30.6		24.0	24.7	
Age at index date, years						
Mean (SD)	74.9 (8.7)	73.3 (10. 6)	–	74.6 (13.3)	74.4 (14.1)	–
40 to <65, %	10.6	17.3	19.5	13.4	13.7	0.8
65≤ to <75, %	33.1	30.2	6.4	30.5	30.0	1.1
75≤ to <85, %	44.2	40.9	6.7	43.0	43.5	1.0
≥85, %	12.1	11.7	1.3	13.0	12.8	0.7
Calendar year of index, [b] %						
2019	18.9	31.5	29.4	25.3	25.3	0.1
2020	36.4	37.2	1.7	36.2	36.6	0.7
2021	35.2	25.1	22.1	30.4	29.9	1.0
2022	9.6	6.2	12.5	8.1	8.2	0.3
Month of index date, %						
January	8.9	9.0	0.2	9.0	8.7	1.0
February	8.0	7.0	3.8	7.0	7.1	0.3
March	9.9	6.7	11.4	8.5	8.4	0.1
April	8.9	6.9	7.4	8.1	8.5	1.4
May	4.7	6.8	9.1	5.4	5.7	1.1
June	6.2	10.5	15.5	7.4	8.1	2.4
July	7.9	9.4	5.1	9.1	9.0	0.4
August	7.5	8.3	3.1	8.4	7.9	1.8
September	8.4	8.3	0.4	8.5	8.0	2.0
October	9.4	9.0	1.6	9.1	9.4	1.1
November	9.2	8.6	2.2	8.9	8.7	0.8

December	10.9	9.6	4.6	10.6	10.6	0.1
COPD treatments in the look-back period, [c] %						
LAMA	7.1	15.8	27.4	11.5	11.6	0.1
LABA	2.0	1.3	5.6	1.6	1.3	2.5
LABA/LAMA	40.7	23.8	36.8	29.5	26.5	6.4
ICS/LABA	13.9	18.9	13.6	18.5	21.2	7.3
ICS/LABA/LAMA	0	0	–	0	0	–
Maintenance therapy status						
No maintenance therapy	36.7	40.9	8.5	39.5	40.0	0.9
LABA or LAMA monotherapy	8.1	15.4	23.0	11.8	11.6	0.5
LABA/LAMA or ICS/LABA dual therapy	55.2	43.7	23.2	48.7	48.4	0.6
ICS/LABA/LAMA triple therapy	0.0	0.0	–	0.0	0.0	–
ICS	27.6	47.5	42.0	41.3	44.1	6.0
OCS	18.2	27.6	22.5	23.2	23.1	0.1
Home oxygen therapy	9.1	7.6	5.4	8.3	8.3	0.0
Hospitalization due to COPD exacerbation in the look-back period, [d] %	5.5	7.6	8.5	7.4	7.0	1.5
Hospitalization due to CAP in the look-back period, [d] %	4.1	4.9	3.5	5.1	4.9	0.8
Comorbidities, [d] %						
Asthma (ICD-10 codes only)	56.9	80.1	–	71.3	60.2	–
Asthma (ICD-10 codes and prescription data)	31.1	76.4	101.8	53.7	53.9	0.5

Myocardial infarction	5.5	5.3	0.8	5.5	6.0	2.2
Congestive heart failure	31.4	33.0	3.5	32.9	33.1	0.4
Cerebrovascular disease	15.2	16.0	2.3	15.9	15.8	0.1
Dementia	0	0	–	0	0	–
Peptic ulcer	26.6	27.6	2.3	26.8	27.6	1.7
Peripheral vascular disease	14.5	13.5	3.0	14.4	14.5	0.5
Connective tissue disease	7.3	8.8	5.4	7.7	8.2	2.0
Diabetes	35.4	36.3	1.8	35.7	36.5	1.6
Anxiety	5.2	6.5	5.3	5.9	6.5	2.5
Depression	5.4	6.7	5.7	5.9	6.2	1.3
BMI [d,e]						
Mean (SD)	22.33 (4.0)	22.41 (4.4)	–	22.41 (6.2)	22.33 (6.0)	–
<18.5, %	9.2	10.5	4.5	10.2	10.2	0.0
≥18.5 to <25, %	33.6	33.6	0.0	34.2	34.5	0.6
≥25, %	12.5	13.9	4.2	13.4	13.7	0.8
Missing, %	44.8	42.0	5.6	42.1	41.6	1.1
Smoking history [d,e], %						
No data	64.0	66.1	4.5	64.5	64.1	0.6
Smoker	36.0	33.9		35.5	35.9	
Lung-function test, [d] %	40.7	37.7	6.1	38.5	39.2	1.3

[a] Weighted mean, SD and proportion were calculated using inverse probability of treatment weighting. [b] Index date is identified between 22 May 2019 and 5 May 2022. [c] LAMA, LABA, LABA/LAMA, and ICS/LABA treatments are identified during 180 days before the index date. [d] Assessment period is identified during 360 days before the index date. [e] Data were available from the hospitalization record.

BMI: body mass index; CAP: community-acquired pneumonia; COPD: chronic obstructive pulmonary disease; FF/UMEC/VI: fluticasone furoate/umeclidinium/vilanterol; ICD-10: International Classification of Diseases 10th revision; ICS: inhaled corticosteroid; LABA: long-acting β2-agonist; LAMA: long-acting muscarinic antagonist; MITT: multiple-inhaler triple therapy; OCS: oral corticosteroid; PS: propensity score; SD: standard deviation.

Table S3. Hospitalization due to CAP among incident users of FF/UMEC/VI or MITT — complete case analysis and PS adjustment without BMI.

	**Complete Case Analysis**		**PS Adjustment Without BMI**	
	**FF/UMEC/VI Incident Users (*n* = 2176)**	**MITT Incident Users (*n* = 2330)**	**FF/UMEC/VI Incident Users (*n* = 3939)**	**MITT Incident Users (*n* = 4017)**
Total patient-years	1163	750	2275	1416
Events	104	79	144	112
FF/UMEC/VI versus MITT unadjusted HR (95% CI)	0.99 (0.74–1.32)		0.92 (0.72–1.17)	
FF/UMEC/VI versus MITT adjusted HR (95% CI)	1.15 (0.80–1.65)		1.05 (0.76–1.44)	

BMI: body mass index; CAP: community-acquired pneumonia; CI: confidence interval; FF/UMEC/VI: fluticasone furoate/umeclidinium/vilanterol; HR: hazard ratio; MITT: multiple-inhaler triple therapy; PS: propensity score.

Table S4. Hospitalization due to CAP among incident users of FF/UMEC/VI or MITT — sensitivity analysis (if COPD is defined on the condition that the patient has a record of prescription of an inhaled COPD medicine in addition to the diagnosis).

	**Complete Case Analysis**		**PS Adjustment Without BMI**		**PS Adjustment with Multiple Imputation for BMI**		**PS Adjustment with Missing-Indicator Method for BMI**	
	**FF/UMEC/VI Incident Users (*n* = 1419)**	**MITT Incident Users (*n* = 1483)**	**FF/UMEC/VI Incident Users (*n* = 2685)**	**MITT Incident Users (*n* = 2586)**	**FF/UMEC/VI Incident Users (*n* = 2685)**	**MITT Incident Users (*n* = 2586)**	**FF/UMEC/VI Incident Users (*n*= 2685)**	**MITT Incident Users (*n* = 2586)**
Total patient-years	843	519	1,710	955	1710	955	1710	955
Events	81	46	108	68	108	68	108	68
FF/UMEC/VI versus MITT unadjusted HR (95% CI)	1.20 (0.83–1.72)		0.98 (0.72–1.32)		0.98 (0.72–1.32)		0.98 (0.72–1.32)	
FF/UMEC/VI versus MITT adjusted HR (95% CI)	1.46 (0.91–2.36)		1.13 (0.73–1.74)		1.14 (0.74–1.76)		1.19 (0.77–1.84)	

BMI: body mass index; CAP: community-acquired pneumonia; CI: confidence interval; COPD: chronic obstructive pulmonary disease; FF/UMEC/VI: fluticasone furoate/umeclidinium/vilanterol; HR: hazard ratio; MITT: multiple-inhaler triple therapy; PS: propensity score.

Table S5. Hospitalization due to CAP among incident users of FF/UMEC/VI or MITT — sensitivity analysis (if the definition of MITT is based on ≥14 days of overlapping prescription periods for three components).

	**Complete Case Analysis**		**PS Adjustment Without BMI**		**PS Adjustment with Multiple Imputation for BMI**		**PS Adjustment with Missing-Indicator Method for BMI**	
	**FF/UMEC/VI Incident Users (*n* = 2176)**	**MITT Incident Users (*n* = 2058)**	**FF/UMEC/VI Incident Users (*n* = 3939)**	**MITT Incident Users (*n* = 3553)**	**FF/UMEC/VI Incident Users (*n* = 3939)**	**MITT Incident Users (*n* = 3553)**	**FF/UMEC/VI Incident Users (*n* = 3939)**	**MITT Incident Users (*n* = 3553)**
Total patient-years	1163	727	2275	1374	2275	1374	2275	1374
Events	104	79	144	112	144	112	144	112
FF/UMEC/VI versus MITT unadjusted HR (95% CI)	0.94 (0.70–1.26)		0.88 (0.69–1.12)		0.88 (0.69–1.12)		0.88 (0.69–1.12)	
FF/UMEC/VI versus MITT adjusted HR (95% CI)	1.08 (0.75–1.55)		0.97 (0.70–1.34)		0.99 (0.72–1.37)		1.00 (0.72–1.38)	

BMI: body mass index; CAP: community-acquired pneumonia; CI: confidence interval; FF/UMEC/VI: fluticasone furoate/umeclidinium/vilanterol; HR: hazard ratio; MITT: multiple-inhaler triple therapy; PS: propensity score.

Table S6. Hospitalization due to CAP among incident users of FF/UMEC/VI or MITT — sensitivity analysis (when the maximum observation period was 1440 days after the index date).

	**Complete Case Analysis**		**PS Adjustment Without BMI**		**PS Adjustment with Multiple Imputation for BMI**		**PS Adjustment with Missing-Indicator Method for BMI**	
	**FF/UMEC/VI Incident Users (*n* = 2176)**	**MITT Incident Users (*n* = 2330)**	**FF/UMEC/VI Incident Users (*n* = 3939)**	**MITT Incident Users (*n* = 4017)**	**FF/UMEC/VI Incident Users (*n* = 3939)**	**MITT Incident Users (*n* = 4017)**	**FF/UMEC/VI Incident Users (*n* = 3939)**	**MITT Incident Users (*n* = 4017)**
Total patient-years	1645	929	3409	1807	3409	1807	3409	1807
Events	125	85	177	124	177	124	177	124
FF/UMEC/VI versus MITT unadjusted HR (95% CI)	1.01 (0.77–1.34)		0.92 (0.73–1.16)		0.92 (0.73–1.16)		0.92 (0.73–1.16)	
FF/UMEC/VI versus MITT adjusted HR (95% CI)	1.16 (0.83–1.64)		1.05 (0.78–1.41)		1.07 (0.79–1.44)		1.07 (0.79–1.45)	

BMI: body mass index; CAP: community-acquired pneumonia; CI: confidence interval; FF/UMEC/VI: fluticasone furoate/umeclidinium/vilanterol; HR: hazard ratio; MITT: multiple-inhaler triple therapy; PS: propensity score.

Table S7. Incidence rates of hospitalization due to CAP among overall users of FF/UMEC/VI or MITT — complete case analysis and PS adjustment without BMI.

	**Complete Case Analysis**		**PS Adjustment Without BMI**	
	**FF/UMEC/VI Overall Users (*n* = 4760)**	**MITT Overall Users (*n* = 5904)**	**FF/UMEC/VI Overall Users (*n* = 8790)**	**MITT Overall Users (*n* = 10,881)**
Total patient-years	2715	2421	5432	4828
Events	234	267	326	356
Unadjusted incidence rate (95% CI) per 1000 patient-years	86.18 (75.74–98.07)	110.28 (97.66–124.53)	60.01 (53.81–66.93)	73.73 (66.40–81.88)
Adjusted incidence rate (95% CI) per 1000 patient-years	227.24 (155.48–332.13)	296.86 (200.38–439.78)	230.32 (165.46–320.59)	295.68 (210.34–415.64)

BMI: body mass index; CAP: community-acquired pneumonia; CI: confidence interval; FF/UMEC/VI: fluticasone furoate/umeclidinium/vilanterol; MITT: multiple-inhaler triple therapy; PS: propensity score.

Table S8. Incidence rates of hospitalization due to CAP among overall users of FF/UMEC/VI or MITT — sensitivity analysis (if COPD is defined on the condition that the patient has a record of prescription of an inhaled COPD medicine in addition to the diagnosis).

	**Complete case analysis**		**PS adjustment without BMI**		**PS adjustment with multiple imputation for BMI**		**PS adjustment with missing-indicator method for BMI**	
	**FF/UMEC/VI overall users (*n* = 4007)**	**MITT overall users (*n* = 5066)**	**FF/UMEC/VI overall users (*n* = 7545)**	**MITT overall users (*n* = 9491)**	**FF/UMEC/VI overall users (*n* = 7545)**	**MITT overall users (*n* = 9491)**	**FF/UMEC/VI overall users (*n* = 7545)**	**MITT overall users (*n* = 9491)**
Total patient-years	2405	2210	4880	4407	4880	4407	4880	4407
Events	210	233	290	314	290	314	290	314
Unadjusted incidence rate (95% CI) per 1000 patient-years	87.31 (76.19–100.07)	105.43 (92.58–120.05)	59.42 (52.93–66.70)	71.25 (63.74–79.65)	59.42 (52.93–66.70)	71.25 (63.74–79.65)	59.42 (52.93–66.70)	71.25 (63.74–79.65)
Adjusted incidence rate (95% CI) per 1000 patient-years	225.26 (149.67–339.04)	264.08 (173.65–401.61)	225.96 (158.64–321.84)	269.36 (187.96–386.02)	222.73 (155.65–318.71)	263.66 (183.13–379.59)	210.51 (146.35–302.79)	247.93 (170.91–359.67)

BMI: body mass index; CAP: community-acquired pneumonia; CI: confidence interval; COPD: chronic obstructive pulmonary disease; FF/UMEC/VI: fluticasone furoate/umeclidinium/vilanterol; MITT: multiple-inhaler triple therapy; PS: propensity score.

Table S9. Incidence rates of hospitalization due to CAP among overall users of FF/UMEC/VI or MITT — sensitivity analysis (if the definition of MITT is based on ≥14 days of overlapping prescription periods for three components).

	**Complete Case Analysis**		**PS Adjustment Without BMI**		**PS Adjustment with Multiple Imputation for BMI**		**PS Adjustment with Missing-Indicator Method for BMI**	
	**FF/UMEC/VI Overall Users (*n* = 4760)**	**MITT Overall Users (*n* = 5592)**	**FF/UMEC/VI Overall Users (*n* = 8790)**	**MITT Overall Users (*n* = 10,358)**	**FF/UMEC/VI Overall Users (*n* = 8790)**	**MITT Overall Users (*n* = 10,358)**	**FF/UMEC/VI Overall Users (*n* = 8790)**	**MITT Overall Users (*n* = 10,358)**
Total patient-years	2715	2415	5432	4813	5432	4813	5432	4813
Events	234	268	326	357	326	357	326	357
Unadjusted incidence rate (95% CI) per 1000 patient-years	86.18 (75.74–98.07)	110.99 (98.30–125.32)	60.01 (53.81–66.93)	74.17 (66.80–82.35)	60.01 (53.81–66.93)	74.17 (66.80–82.35)	60.01 (53.81–66.93)	74.17 (66.80–82.35)
Adjusted incidence rate (95% CI) per 1000 patient-years	228.69 (156.52–334.15)	303.19 (204.46–449.59)	231.44 (166.26–322.16)	300.91 (213.88–423.35)	227.81 (162.71–318.95)	294.55 (208.23–416.65)	220.91 (157.27–310.30)	285.13 (200.47–405.53)

BMI: body mass index; CAP: community-acquired pneumonia; CI: confidence interval; FF/UMEC/VI: fluticasone furoate/umeclidinium/vilanterol; MITT: multiple-inhaler triple therapy; PS: propensity score.

Table S10. Incidence rates of hospitalization due to CAP among overall users of FF/UMEC/VI or MITT — sensitivity analysis (when the maximum observation period was 1440 days after the index date).

	**Complete Case Analysis**		**PS Adjustment Without BMI**		**PS Adjustment with Multiple Imputation for BMI**		**PS Adjustment with Missing-Indicator Method for BMI**	
	**FF/UMEC/VI Overall Users (*n* = 4760)**	**MITT Overall Users (*n* = 5904)**	**FF/UMEC/VI Overall Users (*n* = 8790)**	**MITT Overall Users (*n* = 10,881)**	**FF/UMEC/VI Overall Users (*n* = 8790)**	**MITT Overall Users (*n* = 10,881)**	**FF/UMEC/VI Overall Users (*n* = 8790)**	**MITT Overall Users (*n* = 10,881)**
Total patient-years	4069	3190	8660	6497	8660	6497	8660	6497
Events	305	298	435	407	435	407	435	407
Unadjusted incidence rate (95% CI) per 1000 patient-years	74.95 (66.86–84.02)	93.42 (83.11–105.00)	50.23 (45.67–55.25)	62.65 (56.75–69.16)	50.23 (45.67, 55.25)	62.65 (56.75–69.16)	50.23 (45.67–55.25)	62.65 (56.75–69.16)
Adjusted incidence rate (95% CI) per 1000 patient-years	215.51 (152.21–305.13)	277.96 (194.44–397.34)	207.98 (153.05–282.62)	261.76 (190.72–359.28)	206.64 (151.49, 281.86)	260.43 (189.45–357.99)	198.01 (144.66–271.05)	249.24 (180.39–344.36)

BMI: body mass index; CAP: community-acquired pneumonia; CI: confidence interval; FF/UMEC/VI: fluticasone furoate/umeclidinium/vilanterol; MITT: multiple-inhaler triple therapy; PS: propensity score.

Table S11. Incidence rates of hospitalization due to CAP among incident users of FF/UMEC/VI or MITT — complete case analysis and PS adjustment without BMI.

	**Complete Case Analysis**		**PS Adjustment Without BMI**	
	**FF/UMEC/VI Incident Users (*n* = 2176)**	**MITT Incident Users (*n* = 2330)**	**FF/UMEC/VI Incident Users (*n* = 3939)**	**MITT Incident Users (*n* = 4017)**
Total patient-years	1163	750	2275	1416
Events	104	79	144	112
Unadjusted incidence rate (95% CI) per 1000 patient-years	89.44 (73.71–108.52)	105.30 (84.21–131.68)	63.29 (53.72–74.56)	79.12 (65.61–95.41)
Adjusted incidence rate (95% CI) per 1000 patient-years	182.74 (97.16–343.67)	252.68 (128.85–495.49)	166.30 (91.34–302.76)	238.68 (128.76–442.44)

BMI: body mass index; CAP: community-acquired pneumonia; CI: confidence interval; FF/UMEC/VI: fluticasone furoate/umeclidinium/vilanterol; MITT: multiple-inhaler triple therapy; PS: propensity score.

Table S12. Incidence rates of hospitalization due to CAP among incident users of FF/UMEC/VI or MITT — sensitivity analysis (if COPD is defined on the condition that the patient has a record of prescription of an inhaled COPD medicine in addition to the diagnosis).

	**Complete Case Analysis**		**PS Adjustment Without BMI**		**PS Adjustment With Multiple Imputation for BMI**		**PS Adjustment With Missing-Indicator Method for BMI**	
	**FF/UMEC/VI Incident Users (*n* = 1419)**	**MITT Incident Users (*n* = 1483)**	**FF/UMEC/VI Incident Users (*n* = 2685)**	**MITT Incident Users (*n* = 2586)**	**FF/UMEC/VI Incident Users (*n* = 2685)**	**MITT Incident Users (*n* = 2586)**	**FF/UMEC/VI Incident Users (*n* = 2685)**	**MITT Incident Users (*n* = 2586)**
Total patient-years	843	519	1710	955	1710	955	1710	955
Events	81	46	108	68	108	68	108	68
Unadjusted incidence rate (95% CI) per 1000 patient-years	96.13 (77.24–119.65)	88.58 (66.17–118.57)	63.14 (52.26–76.28)	71.17 (56.03–90.40)	63.14 (52.26–76.28)	71.17 (56.03–90.40)	63.14 (52.26–76.28)	71.17 (56.03–90.40)
Adjusted incidence rate (95% CI) per 1000 patient-years	149.16 (71.11–312.90)	132.20 (59.89–291.80)	142.68 (71.82–283.45)	157.80 (78.66–316.57)	134.84 (67.41–269.74)	146.92 (72.43–298.03)	119.94 (59.15–243.21)	128.01 (61.39–266.94)

BMI: body mass index; CAP: community-acquired pneumonia; CI: confidence interval; COPD: chronic obstructive pulmonary disease; FF/UMEC/VI: fluticasone furoate/umeclidinium/vilanterol; MITT: multiple-inhaler triple therapy; PS: propensity score.

Table S13. Incidence rates of hospitalization due to CAP among incident users of FF/UMEC/VI or MITT — sensitivity analysis (if the definition of MITT is based on ≥14 days of overlapping prescription periods for three components).

	**Complete Case Analysis**		**PS Adjustment Without BMI**		**PS Adjustment With Multiple Imputation for BMI**		**PS Adjustment With Missing-Indicator Method for BMI**	
	**FF/UMEC/VI Incident Users (*n* = 2176)**	**MITT Incident Users (*n* = 2058)**	**FF/UMEC/VI Incident Users (*n* = 3939)**	**MITT Incident Users (*n* = 3553)**	**FF/UMEC/VI Incident Users (*n* = 3939)**	**MITT Incident Users (*n* = 3553)**	**FF/UMEC/VI Incident Users (*n* = 3939)**	**MITT Incident Users (*n* = 3553)**
Total patient-years	1163	727	2275	1374	2275	1374	2275	1374
Events	104	79	144	112	144	112	144	112
Unadjusted incidence rate (95% CI) per 1000 patient-years	89.44 (73.71–108.52)	108.64 (86.85–135.89)	63.29 (53.72–74.56)	81.49 (67.57–98.29)	63.29 (53.72–74.56)	81.49 (67.57–98.29)	63.29 (53.72–74.56)	81.49 (67.57–98.29)
Adjusted incidence rate (95% CI) per 1000 patient-years	182.33 (96.56–344.28)	265.63 (134.58–524.29)	164.90 (90.31–301.09)	249.39 (133.87–464.59)	160.62 (87.49–294.86)	239.59 (127.79–449.23)	158.01 (86.09–290.02)	236.56 (125.24–446.82)

BMI: body mass index; CAP: community-acquired pneumonia; CI: confidence interval; FF/UMEC/VI: fluticasone furoate/umeclidinium/vilanterol; MITT: multiple-inhaler triple therapy; PS: propensity score.

Table S14. Incidence rates of hospitalization due to CAP among incident users of FF/UMEC/VI or MITT — sensitivity analysis (when the maximum observation period was 1440 days after the index date).

	**Complete Case Analysis**		**PS Adjustment Without BMI**		**PS Adjustment With Multiple Imputation for BMI**		**PS Adjustment With Missing-Indicator Method for BMI**	
	**FF/UMEC/VI Incident Users (*n* = 2176)**	**MITT Incident Users (*n* = 2330)**	**FF/UMEC/VI Incident Users (*n* = 3939)**	**MITT Incident Users (*n* = 4017)**	**FF/UMEC/VI Incident Users (*n* = 3939)**	**MITT Incident Users (*n* = 4017)**	**FF/UMEC/VI Incident Users (*n* = 3939)**	**MITT Incident Users (*n* = 4017)**
Total patient-years	1645	929	3409	1807	3409	1807	3409	1807
Events	125	85	177	124	177	124	177	124
Unadjusted incidence rate (95% CI) per 1000 patient-years	76.00 (63.59–90.84)	91.45 (73.50–113.78)	51.92 (44.73–60.28)	68.63 (57.36–82.13)	51.92 (44.73–60.28)	68.63 (57.36–82.13)	51.92 (44.73–60.28)	68.63 (57.36–82.13)
Adjusted incidence rate (95% CI) per 1000 patient-years	170.74 (93.31–312.44)	230.44 (119.58–444.08)	153.95 (87.79–269.95)	216.97 (120.88–389.46)	153.92 (87.67–270.22)	214.21 (119.35–384.49)	147.97 (84.05–260.51)	205.90 (113.59–373.24)

BMI: body mass index; CAP: community-acquired pneumonia; CI: confidence interval; FF/UMEC/VI: fluticasone furoate/umeclidinium/vilanterol; MITT: multiple-inhaler triple therapy; PS: propensity score.

A

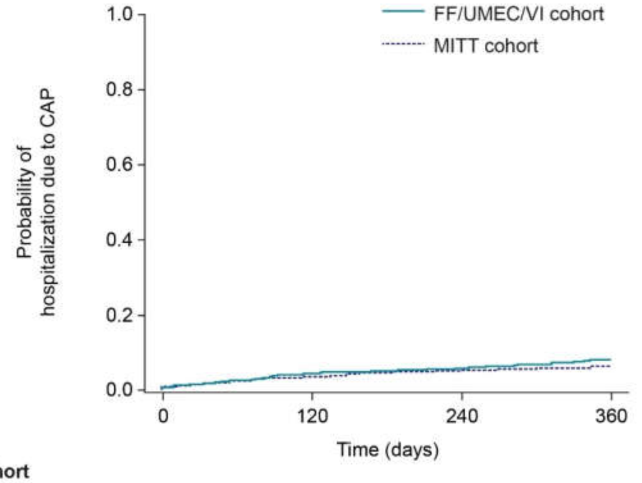

	0	120	240	360
**FF/UMEC/VI cohort**				
Cumulative number of events	0	170	204	238
Number at risk	4473	2671	1713	1249
Cumulative probability	0.000	0.043	0.058	0.080
**MITT cohort**				
Cumulative number of events	0	120	134	138
Number at risk	4509	1091	533	346
Cumulative probability	0.000	0.035	0.053	0.062

B

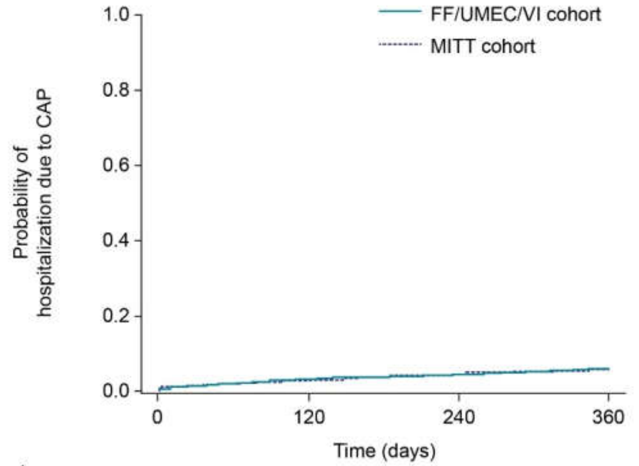

	0	120	240	360
**FF/UMEC/VI cohort**				
Cumulative number of events	0	217	268	315
Number at risk	7892	4974	3343	2512
Cumulative probability	0.000	0.030	0.042	0.058
**MITT cohort**				
Cumulative number of events	0	169	195	207
Number at risk	8011	2292	1169	772
Cumulative probability	0.000	0.028	0.043	0.055

Figure S1. Cumulative incidence of first occurrence of hospitalization due to CAP for incident FF/UMEC/VI and MITT users for (A) the complete case analysis, and (B) when PS is adjusted without BMI.

BMI: body mass index; CAP: community-acquired pneumonia: FF/UMEC/VI, fluticasone furoate/umeclidinium/vilanterol; MITT: multiple-inhaler triple therapy.
